# A Retrospective Review of Resuscitation Planning at a Children’s Hospital

**DOI:** 10.3390/children5010009

**Published:** 2018-01-04

**Authors:** Jean Kelly, Jo Ritchie, Leigh Donovan, Carol Graham, Anthony Herbert

**Affiliations:** 1 Paediatric Palliative Care Service, Division of Medicine, Children’s Health Queensland Hospital and Health Service, South Brisbane, QLD 4101, Australia; jean.kelly@health.qld.gov.au (J.K.); leigh.donovan@health.qld.gov.au (L.D.); 2 Bone Marrow and Transplant Service, Children’s Health Queensland Hospital and Health Service, South Brisbane, QLD 4101, Australia; jo.ritchie@health.qld.gov.au; 3 Behavioural Sciences Unit, School of Women’s and Children’s Health, University of New South Wales, Randwick, NSW 2031, Australia; 4 Children’s Health Queensland Clinical Unit, Faculty of Medicine, University of Queensland, South Brisbane, QLD 4101, Australia; carol.graham@uq.net.au

**Keywords:** resuscitation plan, advance care plan, paediatric palliative care, shared decision-making

## Abstract

Resuscitation plans (RP) are an important clinical indicator relating to care at the end of life in paediatrics. A retrospective review of the medical records of children who had been referred to the Royal Children’s Hospital, Brisbane, Australia who died in the calendar year 2011 was performed. Of 62 records available, 40 patients (65%) had a life limiting condition and 43 medical records (69%) contained a documented RP. This study demonstrated that both the underlying condition (life-limiting or life-threatening) and the setting of care (Pediatric Intensive Care Unit or home) influenced the development of resuscitation plans. Patients referred to the paediatric palliative care (PPC) service had a significantly longer time interval from documentation of a resuscitation plan to death and were more likely to die at home. All of the patients who died in the paediatric intensive care unit (PICU) had a RP that was documented within the last 48 h of life. Most RPs were not easy to locate. Documentation of discussions related to resuscitation planning should accommodate patient and family centered care based on individual needs. With varied diagnoses and settings of care, it is important that there is inter-professional collaboration, particularly involving PICU and PPC services, in developing protocols of how to manage this difficult but inevitable clinical scenario.

## 1. Introduction

There is increasing interest and research around pediatric Advance Care Planning (pACP) [1]. pACP incorporates the wishes of parents or guardians of children with life-limiting or life-threatening conditions. The wishes and preferences of adolescents who have an emerging competence is also important to consider [2]. Advance care planning in children includes consideration of the goals of care at the end of life, including location of care, cultural and spiritual preferences, and organ/tissue donation. It also includes resuscitation planning, which is the focus of this paper [3].

Resuscitation planning refers to the discussions and decisions related to how health care professionals and parents will respond to a child if they deteriorate rapidly. This is often in the context of a cardiac or respiratory arrest. The response at such times would usually include basic life support including cardio-pulmonary resuscitation (e.g., airway support, expired air resuscitation and chest compressions) as well as advance life support (e.g., intubation, mechanical ventilation, administration of medications such as cardiac inotropes, and cardiac defibrillation). In the context of a life limiting condition, particularly if there are concerns the child may not live for longer than 12 months, then it may be appropriate to not provide cardiopulmonary resuscitation (CPR) and to limit or withhold other life sustaining measures such as advance life support. This is particularly in the context of the child’s condition being progressive, with no obvious reversible component of the child’s underlying illness. When a decision has been made to not provide CPR, it is important that other aspects of care such as pain and symptom management are provided, and the dignity of the child is maintained. The patient’s primary pediatrician would usually lead these sensitive discussions around such management with the family often trying to balance hope with reality. Such discussions are becomingly increasingly complex with the emergence of new technologies such as non-invasive ventilation and extracorporeal membrane oxygenation (ECMO) [4,5].

There are a number of barriers to initiating these discussions including time constraints, prognostic uncertainty, disagreement between parents, and clinicians’ difficulty accepting that the patient is not going to recover [6]. Despite the uncertainty in determining prognosis in children, discussion around the issues of resuscitation during end of life care can improve the quality of death and dignity for a child and their family at this difficult time [3].

Parental involvement and shared decision-making regarding treatment of their child throughout end of life is critical as this can influence the family’s bereavement experience [7]. In some studies, parental experience at the end of life is improved if there is comprehensive and sensitive communication from medical staff and an opportunity to talk to the child about death [8]. Those who could acknowledge that there may be a negative outcome earlier and partake in advanced care planning described less distress and an improvement in the quality of life of the child [8,9].

The development of a resuscitation plan (RP) affords the patient and family choice, empowerment and a sense of clarity in communication between clinicians caring for the child [10,11]. In addition, RPs prevent the initiation of invasive procedures with little perceived benefit [11,12]. RPs can be difficult to locate in a medical record outlining the importance of clear documentation to facilitate communication to all involved in the care of the child [13]. In this context, documentation of resuscitation can serve as a quality indicator of shared decision-making with parents (and children where appropriate), and also serves as a clinical tool that can be used at the time of deterioration of a child.

Practice varies between clinicians and ongoing education and evaluation of the approach to resuscitation planning and end of life care is necessary. This study aimed to review both the documentation of resuscitation planning and the ease of access to documentation of discussions relating to resuscitation planning.

## 2. Materials and Methods

The Royal Children’s Hospital (RCH) was a quaternary referral center for pediatric care serving a large area including Queensland and northern New South Wales, Australia, with 20,418 admissions and 166,865 outpatient visits in 2010. A retrospective chart review was performed of the medical records of all children who had been referred to the RCH who died in 2011. A list of deceased patients was obtained from the Health Information Services department and ethics approval was granted by the RCH Human Research Ethics Committee on 20 November 2012 (Reference Number HREC/12/QRCH/224). An audit tool was developed specifically for the purpose of this study and data was collated using Microsoft Excel (Microsoft Corporation, Redmond, WA, USA) and analysed using GraphPad Prism version 7 (GraphPad Software, La Jolla, CA, USA). The RCH closed operations in November 2014 after it merged with the Mater Children’s Hospital to form the Lady Cilento Children’s Hospital.

Data for this audit included the paper-based medical records and the database of the paediatric palliative care service (PPCS), reviewed by a single investigator. Information collected regarding patient characteristics included: age; gender; diagnosis; referral to PPCS; and the cause, date and location of death. Patients were defined as having a life-limiting condition (LLC) using the Directory of Life-Limiting conditions [14]. Parental demographic information was recorded (i.e., marital status, education level and ethnic background). If documentation regarding end of life care, or limitations to treatment was found this was recorded as the “resuscitation plan”. Also recorded was the timing and location of the RP, the individual treatments specified during the discussion, the parent (or guardian) considered to be the decision-maker and whether the individual was considered to be “Gillick competent” [15]. This standard is based on the 1985 decision of the House of Lords in Gillick vs. West Norfolk and Eisbech Area Health Authority, England. The case is binding in England and Wales, and has been adopted in jurisdictions such as Australia, New Zealand and Canada. The original Gillick case related to the prescription of contraception and whether a minor could consent to such treatment without the knowledge or permission of their parent.

A Gillick-competent child has the legal capacity to consent to the provision of medical treatment if they can demonstrate sufficient maturity and intelligence to understand the nature and implications of the proposed treatment, including the risk and alternative courses of actions. There is no fixed age at which a young person is automatically capable of consenting to medical treatment generally, or to specific types of medical treatment. This right to consent is a developing right as the child gains sufficient maturity to make an informed decision. At the same time, the parents’ right to consent decreases, although there will be some overlap.

In some cases, the child’s primary institution was not the RCH and records were either not available or inadequate for inclusion in any analysis. Demographics of patients who suffered from acute trauma resulting in death were recorded but these patients were not included in the present analysis regarding RPs.

Sample means and standard deviations were calculated for the time intervals from resuscitation planning to death in each case in which this information was available. Non-parametric testing was applied using the chi square test to determine if there was statistical significance between proportions. Independent *t*-tests were used when comparing means between groups.

## 3. Results

**Figure 1:**
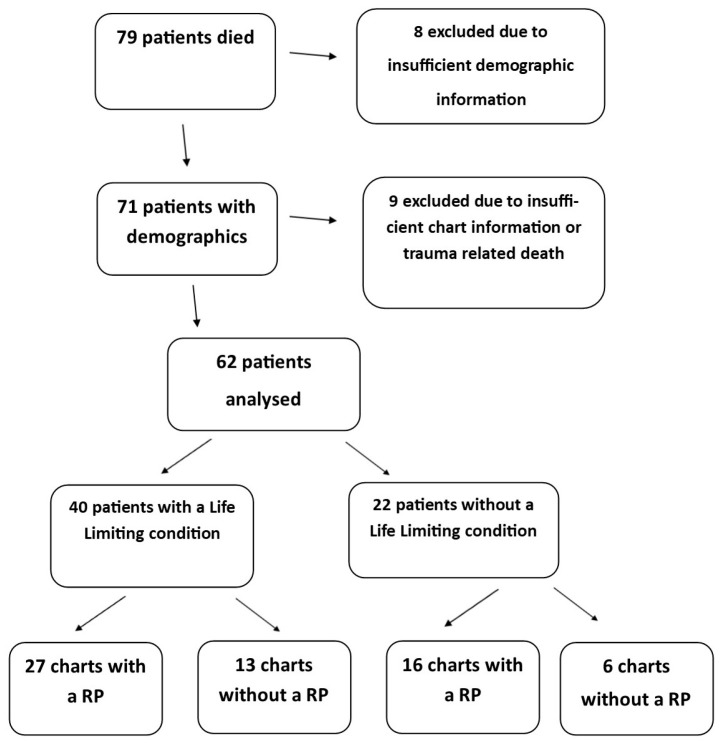
Medical records in which a resuscitation plan (RP) was documented.

Seventy-nine deaths were recorded in the calendar year 2011. Sufficient demographic information was available in 71 of these charts and is outlined in Table 1. Twenty-seven per cent of deaths occurred in the first year of life. The condition with the highest prevalence was malignancy (*n* = 22), followed by neurologic conditions (*n* = 8). Sufficient data for analysis was available in the medical records of 62 patients (Figure 1). Variables that were analysed (i.e., presence of a resuscitation plan, life-limiting condition, referral to palliative care and place of death) are presented in Appendix A.

Of the 62 records available, 43 (69%) contained information related to resuscitation planning. Of these 62 patients, an illness with a poor prognosis or a LLC was diagnosed in 65% of cases (40 of 62). A discussion regarding resuscitation planning was found in the records of 63% (27 of 43) of these patients with a LLC (Figure 1).

The wishes of the child were documented as being considered in only two cases and Gillick competency in three cases. Seven children were aged twelve and over at the time of their death. There was no occasion where treatment was administered which was against the wishes of the parent or guardian.

**Figure 2:**
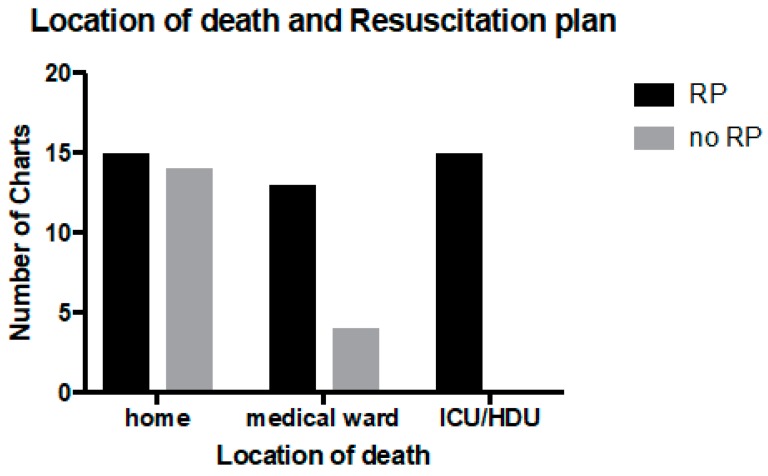
Location of death and presence of a resuscitation plan in 61 patients. ICU: intensive care unit; HDU: high dependency unit.

The largest group of patients died in their own home (23, 37%). Sixteen patients (26%) died in a paediatric intensive care unit (PICU) or high dependency unit (HDU), 15 (24%) died in another medical ward (not PICU or HDU) and 8 (13%) died in an unknown location. The location of death was statistically associated with having a RP (*p* < 0.005), with 100% of patients who died in the PICU having a RP (Figure 2).

**Figure 3:**
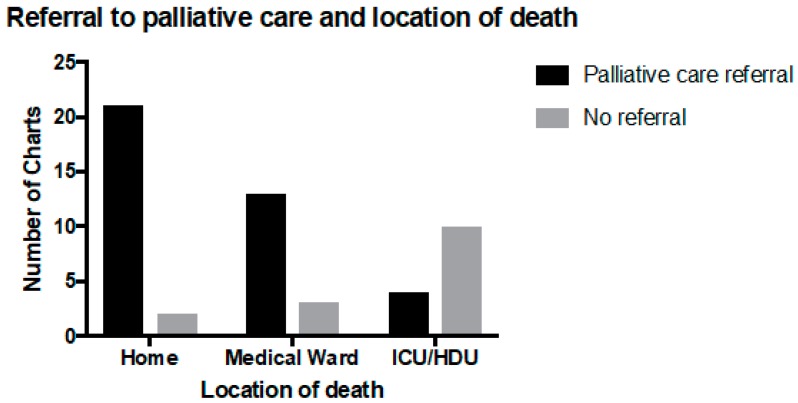
Location of child at time of death and referral to palliative care in 53 patients.

**Figure 4:**
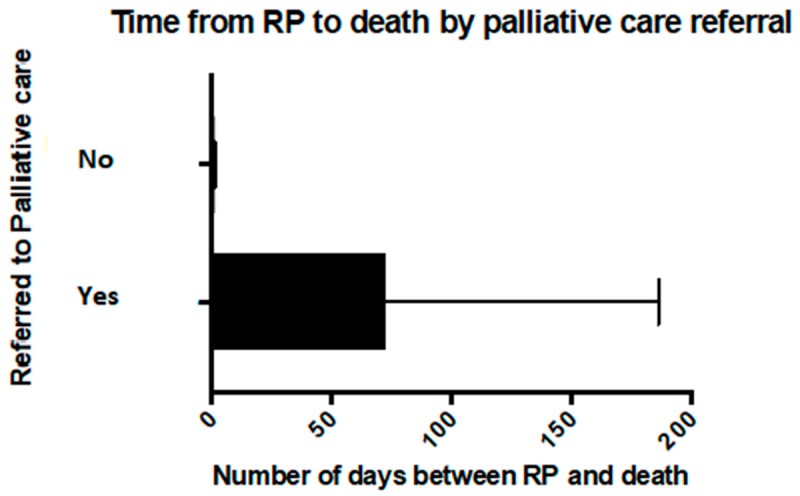
Time from development of RP to death by palliative care referral.

Thirty-nine patients had been referred to palliative care (63%). Of the 16 children who died on the medical ward, 13 (81%) were referred to the PPCS, and among the 15 children who died in the PICU or HDU, four (27%) had been referred to palliative care. Referral to palliative care was significantly associated with dying at home (*p* < 0.05) and outside of the PICU environment (Figure 3) and with a longer time from resuscitation planning to death (*p* < 0.005) (Figure 4). Of the children with a LLC who died at home, 95% had been referred to the PPCS (19 of 20), and 60% (12 of 20) had a RP. Neither a referral to palliative care nor having a LLC was significantly associated with having a RP.

The time from the documentation of a RP to the child’s death ranged from less than 24 h to over one year and was on average 51 days (standard deviation (SD) = 101). However, this included three cases where the RP had been made over 200 days prior to the child’s death (240, 390 and 425 days from RP until death) and when these values were excluded, the average time in days from RP to the death of the child was 25 days (SD = 39). For those patients who died in the PICU or HDU who had a RP, all were documented in the 48-h period before the child died. Overall, discussions relating to the withholding or withdrawing of life sustaining treatment (WWLST) were documented in the 48-h period before death in 37% of cases (*n* = 16).

**Figure 5:**
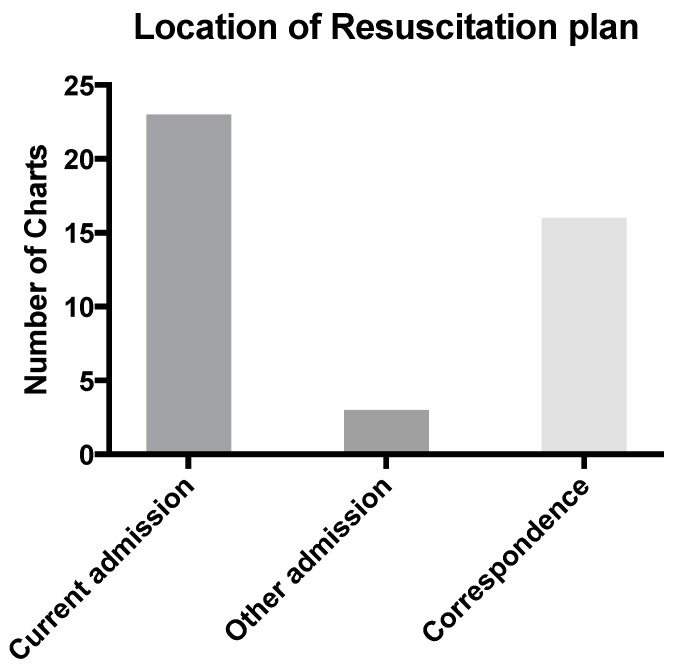
Location of RP.

Only four RPs were easily located. The term “easily located” being considered applicable if it was in a prominent position in the paper-based medical record, highlighted by means of a “tag” or if a distinctive colored ink had been used. Most resuscitation plans were found in the final admission (*n* = 23) with other locations including correspondence (*n* = 16), and prior admissions and notes (*n* = 3) (Figure 5).

## 4. Discussion

### 4.1. Shared Decision-Making

Some clinicians working within paediatric palliative care argue that a focus on RPs is of limited value [16]. There are significant other components to paediatric palliative care (such as symptom management, practical and emotional support) and appropriate spiritual or cultural care that go beyond resuscitation planning. Further, it is argued that the documentation does not truly capture the series of sensitive conversations that may be required for a child and family to experience a dignified death. Nevertheless, a documented RP is a clinical indicator of an important example of shared decision-making related to a very sensitive and difficult aspect of clinical care.

It has been found that early discussion of resuscitation planning is beneficial in a variety of ways, including perceived reduction in pain and suffering, increased psychological support, decreased invasive interventions and importantly, the opportunity for the patient and family to express their wishes and achieve personal goals [8]. This decision also has long-term ramifications, both positive and negative, for other members of the family [7,17,18].

In the current study, there was no care provided that was not consistent with the RP. Similarly, in a children hospice, RPs were followed in all cases except one case where the child underwent unsuccessful resuscitation by a family member who was not a decision-maker [16].

### 4.2. Place of Death

In the current study, RPs were documented in 69% of all patients reviewed. All patients receiving care in the PICU or HDU had RPs documented. This may reflect the practice of shared decision-making and its documentation within the Australian context. One study of 50 consecutive inpatient paediatric deaths at a children’s hospital in Melbourne, Australia found that life-sustaining treatment were either withdrawn or limited prior to death in 84% of cases. There was documented family involvement in the decision-making process in 98% of these cases [13].

In a study of children dying in five different PICUs in the USA, only 56% of patients with life-threatening illness and 64% of patients with life limiting conditions had a formal DNR (Do Not Resuscitate) order in place at the time of death [19]. It was argued that there was a shared understanding of the plan between the multi-disciplinary team within PICU and the family around the process of withdrawal of mechanical ventilation or other life-sustaining therapies. In such a context, it was felt that discussion and documentation of CPR was distracting or irrelevant [19]. Often, DNR orders are established within PICU in the last day or days of life [13,20].

Only 15 patients (15 of 29, 51%) being cared for at home had a RP. This may be due to perception that there is less of a need for such plan in a non-acute healthcare setting. A smaller number of patients (9%) did not have a Do Not Attempt Resuscitation Plan (DNAR) at the time of their death in a study of 207 deaths over a 15-year period within a children’s hospice [16]. It is also possible a RP may have been established in the home by community healthcare professionals (e.g., community nurses or general practitioners) and these had not been communicated back to the hospital. Despite this finding, it is important to develop a RP when home care is being provided, as families may still utilize emergency medical services for various reasons when receiving care at home [21,22].

### 4.3. Palliative Care Involvement

This study found 39 (63%) of patients were referred to palliative care. Sixteen patients (26%) were not referred to palliative care while it was uncertain from the medial record whether a palliative care referral was made in seven cases (11%)—see Appendix A. There are various reasons why a patient may not be referred to palliative care. This would include the patient having an acute and sudden onset life-threatening condition such as sepsis or trauma where there may not be sufficient time for a palliative care referral to be made. In this context, end of life care would appropriately be provided by the PICU. Some children with chronic LLC may not have been referred to the PPCS because their primary team felt they were able to meet the patient’s needs, and a referral to palliative care was not required.

The time between resuscitation planning and death ranged from over one year to less than 24 h, with only two patients having a RP for over one year, and 17 patients having a RP within 24 h of death. The right time to have a RP discussion is influenced by clinical and professional experience, location of care, parental prompts, personal experience, education and religious beliefs [20,23]. As seen with the present study, it appears that when death becomes more of a certainty, discussions regarding WWLST occur more frequently [24]. The development of a RP should ideally occur in a non-crisis environment and afford the family choice, empowerment and a sense of clarity in communication between clinicians caring for their child [10,12].

Patients who were referred to palliative care were more likely to have an earlier documented discussion than patients who were not referred in the current study. The majority of patients who died at home were referred to palliative care. The proportion of patients with a RP who died at home was smaller than that for those who died in hospital. Those who died in hospital, particularly PICU, tended to have their resuscitation plan completed in the final 24 h before the child’s death.

The small sample size in the comparison groups are a limitation in this analysis, but the results are both statistically and clinically significant with all patients who were not referred to palliative care having a RP documented within two days of death. Previous studies have reported an increase in RPs and an increase in time interval between RP and deaths with palliative care and advanced care team consults [3,25]. Wolfe et al. have described early referral to palliative care and instigation of resuscitation planning as markers of quality end of life care [3]. It is likely that those who were not referred to palliative care had a more acute presentation or unpredictable trajectory [19]. However, 65% of patients in the current study had a pre-existing diagnosis associated with a poor prognosis. This suggests opportunities to refer to palliative care earlier in the course of the disease trajectory for some children.

In addition to other components of holistic palliative care (such as addressing goals of care, symptom management and psychosocial support), discussions of prognosis and resuscitation discussions are more likely to occur in children who have received a palliative care consultation [26]. Children who receive a palliative care consultation are more likely to have a DNR order in place for a longer time before death (six versus two days) [27]. Death was also more likely to occur outside of the intensive care environment [27]. The current study supports such findings and extends into the non-cancer and homecare context.

### 4.4. Role of Documentation

Documentation and ease of access of RPs are essential for the health care team to communicate plans to each other and to relieve some stress from the child’s caregivers [8]. Locating documentation regarding RPs was a challenge in the current study and has been reported elsewhere [13]. RPs were not filed in a consistent place in the current study. The inclusion in this study of a large number of patients who died at home has highlighted the role of the RP as a tool, which can communicate the patients’ and parents’ wishes to a variety of service providers [21,22]. The number of clinicians parents encounter during an acute admission to hospital can be overwhelming [11]. In this context, it is helpful if staff can locate a RP readily within the patient’s medical record.

A RP template can serve as a helpful clinical tool. Firstly, it can foster a logical sequence of clinical reasoning—see Paediatric Acute Resuscitation Plan (PARP) in supplementary materials (Figure S2). This can include clinical assessment and decisions relating to treatments that will be provided and those that will be withheld or limited. The form can also encourage documentation of discussion with key decision-makers such as the parent. The form can also prompt health professionals to use clear and compassionate language with families, so they feel supported in this process [12]. In this context, the form can allow both a personalized approach to care, whilst at the same time minimizing unhelpful variation in practice and documentation [10,28]. Finally, such a form can serve as an audit tool when examining practices such a resuscitation planning and advance care planning within paediatrics.

With a move to the use of electronic medical records, such a form can be readily scanned into the medical record. It is possible to place an alert that such a plan exists, including on what date the plan was made. The form can also pop-up as an initial key document when, for example, a “clinical notes” tab is clicked. In the future, we hope to establish a clearly marked “Advance Care Planning” tab where both a resuscitation plan and an advance care plan can be found. The form can also be scanned and forwarded on to the Ambulance Service. In Queensland, the ambulance service will use this form as a basis for their own resuscitation plan. When the ambulance is called to the patient’s address, the paramedics will be notified that a resuscitation plan is in place for one of the residents at that address. We also encourage the parents to hold a printed version of the form that they can present to emergency staff (both paramedics and those working within the emergency department) at the time of presentation.

A further development would be having the form present within the electronic medical record as a template upon which the health professional can fill in the details by typing rather than handwriting. While the use of the PARP is encouraged, other forms of clear documentation or correspondence are permissible as an alternative. A similar process for establishing an alert and liaison with the ambulance service is still possible in this context.

### 4.5. Limitations

As with any chart review, data was limited to the information charted by the healthcare professionals. Limitations included incomplete charting, differences in documentation style and procedures, location of documents and missing information. Additionally, some charts had discontinuity in terms of location of care for patients, potentially resulting in incomplete chart information. The current audit identified if the decision-maker (usually the parent) was documented and also whether the young person had the ability to provide consent. Further improvement would be to audit whether there was a documentation of the discussion between the parents and the health professionals as a marker of shared decision-making. Furthermore, it would be helpful to also audit whether young people had Gillick competence, or alternatively if they had developmental disability precluding involvement in medical decision-making.

In addition to observing the place of death of the child, it would also be helpful to determine if the child and family had expressed a wish for where they would die. It would then be possible to determine how many patients died in their preferred location of death. Such information is more likely to be contained in an Advance Care Plan rather than a Resuscitation Plan. Some research suggests that it is not necessarily the location of death, or whether the child died in the preferred location of death, but rather if the family were given the options and choices around where their child could die [29].

## 5. Conclusions

This study has suggested a number of improvements in practice. This included prominent placement of RP within the medical record and improved documentation of resuscitation plans for those who die at home. Documentation of the shared decision-making process between health professionals and families in relation to RP is also important. This would include assessment of the competency of the older child to be involved in such discussions and decision-making. Setting of care and sub-specialty involvement (e.g., palliative care and/or intensive care) also impacted RPs. Patients who died in PICU were more likely to have a resuscitation plan in place compared to those who died at home. Those patients involved with palliative care were more likely to have their resuscitation plan developed more than 48 h before they died. The use of a template to document resuscitation plans can be an effective clinical and communication tool for families and clinicians at the time of deterioration.

## Figures and Tables

**Table 1:** Patient characteristics.

**Gender of Child**		***n* = 71**
Male		36
Female		35
**Age of Child**		***n* = 71**
0–3 months		8
3–6 months		5
6–12 months		6
1–5 years		12
5–10 years		28
>10 years		12
**Diagnosis**		***n* = 71**
Oncology	Brain Tumour	10
	ALL	4
	PTLD	2
	Other malignancy *	6
Neurological		8
Congenital		7
Chromosomal abnormalities		6
Infection		4
Metabolic		4
Prematurity		4
Unknown		4
Meningitis		3
Accident		3
Other		3
**Parent Demographics**		***n* = 71**
Marital Status	Married	47
	Single	0
	Separated/Divorced	15
	Foster care	2
	Unknown	7
Parent Education	Year 12 or less	12
	Tertiary	8
	Trade	6
	Unknown	45
Parent Ethnicity	Caucasian	43
	Aboriginal or Torres Strait Islander	1
	Other	11
	Unknown	15

ALL: acute lymphoblastic leukaemia; PTLD: post-transplant lymphoproliferative disorder. * Other malignancy includes: sarcoma, ovarian tumour, Wilms tumour, hepatoblastoma, rhabdoid tumour and metastatic adrenocortical carcinoma.

## Supplementary Materials

The following are available online at www.mdpi.com/2227-9067/5/1/9/s1, Figure S1: Audit form, Figure S2: Paediatric Acute Resuscitation Plan.

## Appendix A

**Table A1:** Frequency of analysed variables (62 patients).

**Resuscitation Plan**	Yes	43 (69%)
	No	19 (31%)
**Life Limiting Condition**	Yes	40 (65%)
	No	22 (35%)
**Palliative Care**	Yes	39 (63%)
	No	16 (26%)
	Unknown	7 (11%)
**Place of Death**	Home	23 (37%)
	Medical ward	16 (26%)
	Paediatric Intensive Care Unit/High Dependency Unit	15 (24%)
	Unknown	8 (13%)
