# Supplementary material for: A Retrospective Review of Resuscitation Planning at a Children’s Hospital
Source: Children (Basel). 2018 Jan 4;5(1):9. doi: 10.3390/children5010009 (PMC5789291; doi:10.3390/children5010009)
Supplement: Supplementary file 1 [file children-05-00009-s001.zip › Figure 1. Resuscitation Audit Tool.pdf]

Queensland Government

Children's Health Queensland

PARP Audit

(Affix patient identification label here)

URN:

Family Name:

Given Names:

Address:

Date of Birth: Sex: ☐ M ☐ F ☐ I

I Patient Demographics

Diagnosis:

Cause of death:

Date of death: / /

Location of deceased:

II Parental Demographics

Marital status

☐ Married or in a stable relationship ☐ Separated / divorced ☐ Single parent ☐ Foster care

Education level

☐ Tertiary ☐ Trade or Certificate ☐ Completed school ☐ Not known

Background

Ethnicity: ☐ Caucasian ☐ ATSI ☐ Other CAL

III Referral to Palliative Care

Referral to Palliative Care Service: ☐ Yes ☐ No

Referral source to Palliative Care: ☐ Tertiary Care Team ☐ Primary Care Team ☐ Other

IV Resuscitation Plan

Did patient have a resuscitation plan? ☐ Yes ☐ No **Date resuscitation plan was made:** / /

V If Patient Died in Hospital

Final ward

☐ Medical ☐ Surgical ☐ HDU/ICU

Transferred from

☐ Regional / rural hospital ☐ Regional / rural home location ☐ Tertiary hospital ☐ Urban home location

Duration of admission

☐ <24 hrs ☐ 24 - 48 hrs ☐ Up to 1 week ☐ Up to 1 month

☐ Up to 3 months ☐ 3 months - 1 year ☐ >1 year

During final admission was there a:

MERT ☐ Yes ☐ No

Resuscitation attempt ☐ Yes ☐ No

Transfer to ICU/HDU ☐ Yes ☐ No

Death within:

☐ 24 Hours ☐ 1 Week

☐ 48 - 72 Hours ☐ >1 Week

Queensland Government

Children's Health Queensland

PARP Audit

(Affix patient identification label here)

URN:

Family Name:

Given Names:

Address:

Date of Birth: Sex: ☐ M ☐ F ☐ I

VI Resuscitation Planning

Documentation	Provide	Don't provide	Not mentioned
MERT Call			
CPR			
Defibrillation			
Date of death			
Ventilation: ☐ Intubation			
☐ Non-invasive ventilation			
ICU Admission (or HDU)			
Comfort measures / palliative support			
Blood Products			
IV fluids			
Antibiotics			
Medication Review			
Cardiac drugs			
Nutrition			

Is there documentation to provide other active life sustaining measures? ☐ Yes ☐ No

Is there documentation to withhold or withdraw other life sustaining measures? ☐ Yes ☐ No

Has any treatment been provided that has been requested to be not provided? ☐ Yes ☐ No

Patient choices documented ☐ Yes ☐ No

Ability to consent / Gillick competence ☐ Yes ☐ No

Decision maker documented ☐ Yes ☐ No

Involvement of Child Safety ☐ Yes ☐ No

VII Documentation

If Resuscitation Plan is documented, where in the chart was it documented?

Front of Chart ☐ Yes

Current admission ☐ Yes

Correspondence ☐ Yes

Other admission ☐ Yes

Was Resuscitation Plan easily identified? ☐ Yes ☐ No

Clinical assessment documented? ☐ Yes ☐ No

Days from Resuscitation Plan documented to death: ....
