# Supplementary material for: A Retrospective Review of Resuscitation Planning at a Children’s Hospital
Source: Children (Basel). 2018 Jan 4;5(1):9. doi: 10.3390/children5010009 (PMC5789291; doi:10.3390/children5010009)
Supplement: Supplementary file 1 [file children-05-00009-s001.zip › Figure 2. Paediatric Acute Resuscitation Plan.pdf]

Queensland Government

PAEDIATRIC Acute Resuscitation Plan (PARP)

For patients under the age of 18 years at risk of an acute deterioration

Facility: ....

(Affix identification label here)

URN:

Family name:

Given name(s):

Address:

Date of birth: Sex: ☐ M ☐ F ☐ I

Decisions about appropriate treatment options must be made in the best interests of the child. Children capable of understanding their situation should be involved in all discussions about their future health care.

- **This PARP form is for use in Queensland Health hospitals and replaces NFR Orders.**
- **For legal reasons, this PARP form is specifically designed for patients under the age of 18 years. For patients over the age of 18 years, use an *Acute Resuscitation Plan (ARP)* form *(SW065)*.**
- **The Quick Guide attached to this form contains important information and should be read prior to completing the form.**
- **If there is insufficient room on this form to record information, cross-reference with the progress notes.**

1. Clinical assessment

Record details/assessment of relevant medical conditions relating to the child's health and wellbeing. A second opinion from a more experienced medical officer should be sought where diagnosis/prognosis is uncertain.

Diagnosis:

2. Resuscitation management plan

If an acute deterioration or critical event occurs, it is clinically indicated to:

Provide e.g. oxygen, intravenous access, airway suctioning, nasogastric tube insertion, non-invasive ventilation

Not provide* e.g. nasogastric tube insertion, non-invasive ventilation, cardiac drugs (e.g. inotropes)

Has a referral been made to palliative care? ☐ Yes ☐ No

There is further documentation in the progress notes on the following dates:

CPR ☐ **Provide** ☐ **Do not provide**

A decision not to provide CPR does not limit other treatment or care

***In the event of acute deterioration, supportive care should be provided with priority given to comfort and relief of distressing symptoms. This should include pain relief, seizure management and sedation as is clinically appropriate. This also includes the provision of privacy with emotional, spiritual and cultural support as required.**

Form continues over page

DO NOT WRITE IN THIS BINDING MARGIN

v10.00 - 09/2016
Product Code: 4327411
SW267

PAEDIATRIC ACUTE RESUSCITATION PLAN (PARP)

General

- Although not specifically identified, this guide applies to all biological age groupings of patients under the age of 18 years, including neonates and adolescents. The PARP form uses the term child and children very generally to cover this biological spectrum.
- The PARP form should be completed where it is reasonably expected that a child may suffer an acute deterioration or critical event in the foreseeable future and require resuscitation planning. It is recommended that resuscitation planning be initiated as early as practicable to avoid decisions being made in a crisis.
- Patients for whom a PARP form would be appropriate include children who are terminally ill and/or are expected to die within 12 months.

Best interests

- "Best interests" involves weighing the benefits, burdens and risks of treatment, in order to achieve the best possible outcome for the child.
- Assessing the best interests for a child is a careful and thoughtful balancing exercise. While clinical considerations are important, there are other significant elements that may be involved in the decision-making process. Assessing the child's best interests includes taking account of physical matters (e.g. not suffering or being in pain, and minimising distress), social and emotional factors (e.g. enabling interactions with others, feeling safe, and the ability to contribute to relationships) and spiritual and cultural considerations.
- There should be a realistic element to upholding a child's best interests - this may involve balancing competing interests.
- Each child's best interests are unique to them and therefore require consideration on a case-by-case basis.

Legal considerations

- Under the law, medical officers are under no obligation to offer, provide or continue treatment that on balance would have the potential to cause harm and offer no benefit to the patient (i.e. futile).
- The law also requires a collaborative approach among health providers, patients and decision-maker(s) about decisions regarding life-sustaining measures. Appropriate documenting of this is good medical practice. The PARP form prompts this approach.
- A PARP form is a clinical record and does not in itself give consent to provide, withhold or withdraw life-sustaining measures. Legal authority comes from obtaining consent to the *Resuscitation management plan* from the appropriate decision-maker(s).
- Obtaining consent in relation to life-sustaining treatment is a two-step process: first, determine if the patient is Gillick competent for the decision that is required and is therefore the appropriate decision-maker (see Gillick competence); second, if they are, assess whether they are making decisions in their best interests.
- A Gillick-competent child can consent to the provision of life-sustaining treatment. However, they cannot refuse life-sustaining treatment if providing that treatment would be in their best interests. A treatment refusal may be overridden by the Court.
- If the child is not Gillick-competent, the law expects the child's parents or decision-maker(s) to make decisions in the child's best interests.
- The law requires informed consent through a discussion(s) about available end-of-life treatment and care options. When active treatments are no longer appropriate, this should be sensitively explained (in specific or broad terms) to the child's decision-maker(s) and the child if appropriate.
- In emergency situations, while all reasonable efforts should be made to obtain consent, it may be inappropriate to continue to maintain life while attempts are made to obtain consent. Emergency situations are characterised by the need for an immediate decision to be made about maintaining the life and health of a patient.
- Medical treatment should never be withheld merely on the grounds that it is easier to withhold treatment than to withdraw treatment which has been commenced.
- Blood transfusions can be administered to patients under the age of 18 years where consent has been refused or can not be obtained. Under the *Transplantation and Anatomy Act 1979*, two medical officers must consider the transfusion to be necessary to preserve the child's life.
- Legal protections and indemnity are provided to staff who comply with Queensland Health policy on the withholding and withdrawal of life-sustaining measures.

Gillick competence

- A Gillick-competent child has the legal capacity to consent to the provision of medical treatment if they can demonstrate sufficient maturity and intelligence to understand the nature and implications of the proposed treatment, including the risks and alternative courses of actions.
- There is no fixed age at which a young person is automatically capable of consenting to medical treatment generally, or to specific types of medical treatment. This right to consent is a developing right as the child gains sufficient maturity to make an informed decision. At the same time, the parents' right to consent decreases, although there will be some overlap.
- Using the Gillick test of maturity and understanding, a child may be able to consent to straightforward, relatively risk-free treatment, but not necessarily to more complex or high-risk treatment, or to the withholding or withdrawal of life-sustaining treatment, given its serious consequences. It is harder to meet the Gillick test for more significant health decisions.
- Because of the critical nature of decisions around life-sustaining measures, Queensland Health's policy position is that even if the child is Gillick-competent, parents or persons with decision-making authority must be involved in all discussions.
- A medical officer, supported by the health care team, has the responsibility of assessing whether a child is Gillick-competent.

Dispute resolution

- At times, a decision-maker may want to refuse consent to the provision of life-sustaining medical treatment due to personal beliefs (e.g. a belief in alternative remedies to the exclusion of other treatments) or religious reasons (e.g. a Jehovah's Witness refusing a blood transfusion).
- Also, a decision-maker may request or demand treatment not considered by the health care team to be in the child's best interests. This may take the form of a refusal to consent to the withholding or withdrawal of life-sustaining medical treatment. There is no legal or ethical obligation to accede to demands for futile medical treatment.
- The above situations are likely to be indentified when the refusal, request or demand differs from the clinical decision documented in the *Resuscitation management plan*.
- The health care team should make all efforts to explain why the proposed treatment plan is in the best interests of the child. If the decision-maker(s) disagrees with the treatment plan, a second opinion should be offered (from a more experienced clinician, either Queensland Health or external).
- If initial dispute resolution attempts are unsuccessful in what would be considered a reasonable timeframe, the medical officer must refer the matter to the hospital executive. At this point, consideration can be given to applying to the Court for consent.
- Almost all dispute resolution is successful, and it will be rare for the Court to become involved.

Communication

- An essential part of good medical practice is effective communication. This includes:
  - » actively involving the child's decision-maker(s) in the proposed treatment plan;
  - » where it is appropriate to do so, involving the child in advance care planning discussions at their level of development;
  - » asking for and respecting the views of both the decision-maker(s) and the child, where appropriate;
  - » responding to concerns and preferences for the child's future health care;
  - » incorporating cultural and spiritual values, as far as practicable; and
  - » allowing sufficient "acceptance time" for the parents (and the child where appropriate) to absorb the information and reach their decisions by taking their own unique circumstances into account.
- Open, honest and sensitive conversations about available treatment options are vital when any patient is diagnosed with a life-threatening illness or condition. However, it should be acknowledged that the child's parents and some members of the health care team may find it difficult to put aside the emotional distress associated with limiting active medical treatment. See the *Implementation Guidelines, Part 2* for conversation tips, e.g. delivering bad news.
- It should be carefully explained that whatever clinical decisions are made, the child's condition will be monitored to ensure that they are kept comfortable and, as far as possible, free from pain and other distressing symptoms.
- The health care team should support parents who feel pressured by others to insist on or refuse particular investigations or treatment.

Quick guide to completing a Paediatric Acute Resuscitation Plan (PARP)

Remove these instructions before filing this PARP form. It is recommended that the original form be filed at the front of the patient's medical record, but individual facilities can decide on the most prominent place to file the form.

This Quick Guide should be read in conjunction with the *Withholding and Withdrawing Life-Sustaining Measures Policy* and *Implementation Standard,* and the *Implementation Guidelines – End-of-life care: decision-making for withholding and withdrawing life-sustaining measures from patients under the age of 18 years* – Part 1 and Part 2.

Section 1. Clinical assessment

- Record the patient's relevant medical conditions and ensure cross-referencing with the progress notes. This may include clinical reasons why resuscitation planning is necessary.
- If there are doubts or uncertainties about the patient's medical condition, a second opinion should be obtained.

Section 2. Resuscitation management plan

- Record the treatment and care that should, and should not be, provided. Examples given on the PARP form are for illustration only and do not substitute for clinical judgement.
- This is the section that will most often be referred to if an acute deterioration occurs. The instructions in the plan need to be clear and unambiguous to allow other members of the health care team to act on these instructions, and to document accordingly.
- If the attending clinician does not agree with the instructions at the time a decision is required, they must use their clinical judgment to determine the appropriate course of action. In these situations, the clinician must be able to defend their judgment, and document their reasons for not following the instructions.
- Patients may still benefit from a range of treatments and therapies that contribute to quality end-of-life care.
- Completion of this section does not exclude the provision of other treatments which are not specifically mentioned (e.g. palliative therapies, management of pain, suffering and discomfort).
- See *Legal considerations* for information about blood transfusions.

Section 3. Consenting details

- Except in emergency situations, consent must be obtained to act on the *Resuscitation management plan.*
- Consent from the parent(s) or person(s) with decision-making authority must be given in the best interests of the child.
- A child's parent(s) is usually their decision-maker. Sometimes a parenting order issued by the Family Court will determine that a particular parent is the decision-maker. However, parents may be unwilling, unable or not appropriate to contribute to the decision-making process. In these cases, a person with parental authority, based on appointment as a guardian under a will, or under a guardianship order or child protection order, will become the child's decision-maker. If there are doubts, contact the District Child Protection Officer or obtain legal advice.
- A child may be able to be their own decision-maker if they are Gillick competent. See *Gillick competence.*
- Consent can be verbal. Document this on the PARP form or in the patient's progress notes. No signature is required from the decision-maker.
- Although they may not be capable of providing consent, the patient should be involved in treatment decisions if they are able to do so. See *Communication.*

Section 4. Clinician authorisation

- A paediatrician or senior medical officer should complete and sign the PARP form.
- In limited circumstances (e.g. in remote communities), it may be appropriate for a more junior doctor to complete and sign the form. In these circumstances, the PARP form must be authorised by the most senior medical officer available (this can be done over the phone, by fax or email). Note that this carries an element of risk for the health care team involved.
- If the PARP form is not fully completed/signed/authorised, and the patient suffers an acute deterioration or critical event, attending clinicians are required to exercise their clinical judgement based on the circumstances and document this.
- Record recommendations for monitoring and review. Long-term PARP forms should be regularly reviewed as part of good medical practice.
- It may also be appropriate to list other clinicians involved in the care of the patient, and/or the development of the PARP form, as well as clinicians who have been provided with a copy of the PARP form.
- A senior medical practitioner/paediatrician must be involved in all decisions to withhold/withdraw medical treatment.

Voiding the PARP form

- If changes are required or the form has lapsed, it must be marked as void under the authority of a paediatrician or senior medical officer.
- To void the form, draw two lines diagonally across the front and back pages, write 'VOID' between the lines and sign and date this notation. Retain the voided PARP form in the patient's medical record.
- A paediatrician or senior medical officer is responsible for deciding whether an existing PARP form should be voided as a result of changes and whether a new PARP form is required.

Patient transfers and copies

- If a patient is being transferred to another facility, a copy of the PARP form (active, voided or lapsed) should be accompanied by a PARP Cover Sheet.
- The original PARP form must be retained in the medical record.
- Record the contact details of the transferring facility, person or team in the space provided on the Cover Sheet. This information may assist the receiving team or facility to develop appropriate resuscitation planning for the patient.
- Non-Queensland Health facilities are responsible for following their own processes and procedures for documenting or acting on resuscitation planning decisions. This includes the Queensland Ambulance Service while the patient is in transit.
- It is recommended that the patient's GP receive a copy of the patient's PARP form (active, voided or lapsed) for their records.

For further information and resources, contact Access Improvement Service:

Email: QHclinicalethics@health.qld.gov.au

Website: www.health.qld.gov.au/advance-care-planning/

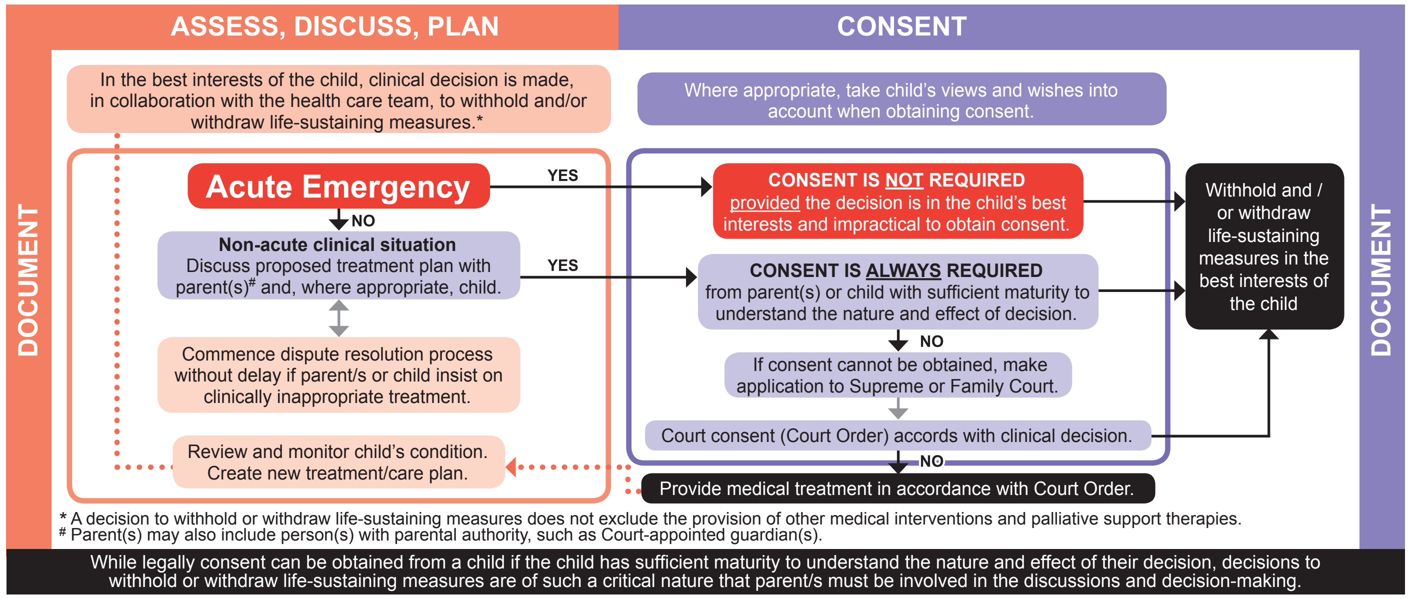

Family name:	Given name(s):	URN:

3. Consenting details

For patients under 18, in the majority of cases the law gives a parent(s)/person(s) with decision-making authority the ability to agree to treatment in the best interests of the child. A child may make decisions about their own medical treatment if they are capable of understanding its significance (see *Gillick competence* in the Quick Guide for further detail).

Details of consenting discussions with parent(s) or person(s) with decision-making authority:

Details of discussions with the child* (if applicable):

* Because of the seriousness of decision-making around life-sustaining measures, a parent(s)/ person(s) with decision-making authority must be involved in the decision-making process, even if the patient (under 18 years) is competent to make decisions on their own behalf.

Details of decision-maker(s): e.g. parent(s) or person(s) with decision-making authority. Must include name(s), contact phone number(s), relationship to child, etc.

☐ Parent(s)

Details:

☐ Court order ☐ Legal guardian ☐ Other

Details:

4. Clinician authorisation

This PARP form remains valid:

☐ For this admission

☐ Until date: / /

☐ For this and subsequent admissions

Medical officer's name:

Medical officer's signature: **Date:** / /

Authorising medical officer's name*, if applicable:

Dr

* A senior medical practitioner/paediatrician must be involved in all decisions to withhold/withdraw medical treatment.

Recommendations for monitoring and review, if applicable:
e.g. will the PARP apply during planned surgery?

Other clinicians involved in the care of the child, the development of this PARP form, and/or provided with a copy:
e.g. GPs, paediatricians, allied health and nursing professionals

If changes are required, this form must be voided and a new PARP form completed

DO NOT WRITE IN THIS BINDING MARGIN
